# Increased level of fibrinogen chains in the proteome of blood platelets in secondary progressive multiple sclerosis patients

**DOI:** 10.1111/jcmm.14244

**Published:** 2019-03-05

**Authors:** Michal Bijak, Alicja Olejnik, Bozena Rokita, Agnieszka Morel, Angela Dziedzic, Elzbieta Miller, Joanna Saluk‐Bijak

**Affiliations:** ^1^ Faculty of Biology and Environmental Protection, Department of General Biochemistry University of Lodz Lodz Poland; ^2^ Chemistry Department, Institute of Applied Radiation Chemistry Lodz University of Technology Lodz Poland; ^3^ Department of Physical Medicine Medical University of Lodz Lodz Poland; ^4^ Neurorehabilitation Ward, III General Hospital in Lodz Lodz Poland

**Keywords:** blood platelets, fibrinogen, multiple sclerosis, proteome, thrombosis

## Abstract

Epidemiological studies indicate a high risk of stroke, heart failure and myocardial infarction in patients with multiple sclerosis, especially in its secondary progressive (SPMS) phase. Some ischaemic events are directly associated with abnormal platelet functions and their prothrombotic activity. Recent reports, including this study, confirm the increased activation of circulating platelets in SPMS, and also show increased platelet reactivity, among other responses, as well as strong aggregation. In this current study, we conducted a comparative analysis of the platelet proteome in SPMS patients and in healthy controls, to demonstrate the quantitative and qualitative differences likely to affect functional changes observed in SPMS. During densitometry evaluation of 2‐D fluorescence difference gel electrophoresis, we observed differences between the electrophoretic patterns of SPMS platelets and the control samples. To determine a detailed characterisation of the proteome changes in the SPMS patients’ blood platelets, in the next stage, we performed mass spectrometry of selected spots and indicated the increased presence of four proteins (fibrinogen, α‐2 macroglobulin, septin‐14 and tubulin β‐1 chain). The most important of these is the increased amount of prothrombotic protein, fibrinogen, which seems to confirm the accuracy of the imaging and potentially explains the increased risk of platelet‐origin thrombotic events. This study provides new knowledge of the potential existence of the molecular mechanisms responsible for the acceleration of the platelet pro‐coagulant function in SPMS. This can help to identify new targets for therapy, which can then be used not only in the second stage of the disease.

## 1 INTRODUCTION

Multiple sclerosis (MS) is an immune‐mediated, demyelinating disease of the central nervous system (CNS). The aetiology of MS remains unclear, but it is believed that this disease has an autoimmune origin with predominant simultaneous inflammatory and neurodegenerative processes. The essence of MS lies in the formation of inflammatory plaque and the degradation of nerve myelin within the white and grey matter of the CNS, leading to the impairment of neurotransmission, and consequently to the occurrence of neurological symptoms.^1^ On pathological and clinical grounds, MS is a heterogeneous disease, and therefore different pathophysiological pathways can be activated in different MS patients, to initiate and perpetuate the devastating pathologic process.^2^

Four main subtypes of the disease are recognized as a result of MS's heterogeneous neurological symptoms. The most common is the first phase of MS, relapsing‐remitting (RRMS), in which the disease fluctuates between periods of inflammation/demyelination and remission. Sometimes, the disease starts with a primary progressive stage (PPMS), which is characterized from the beginning by progression without any remission states. After several years of the disease's duration, when the axons have lost their ability to regenerate, in more than 80% of cases RRMS turns into a secondary progressive stage (SPMS), in which the patient suffers irreversible progressive disability. The least widespread of MS subtypes is progressive‐relapsing (PRMS).^3^

The nature of the pathophysiology of MS is very complex and almost always involves multiple types of cells. It has been well established that various immune cells, such as the T helper lymphocytes (Th1, Th17), B cells and macrophages, are involved in the pathogenesis of MS.^4^ Blood platelets have also been suggested as contributing to the development of MS. The interaction of blood platelets and immune and endothelial cells is responsible for the disruption of the blood‐brain barrier, which leads to infiltration of lymphocytes, and further, to the formation of inflammatory and demyelinating lesions in CNS.^5^, ^6^

It has been proved that blood platelets are an important factor in the development of inflammation in the early phase of MS.^7^ The major role of blood platelets in the development of inflammatory response comes from the ease with which they activate and adhere to inflamed endothelial cells or protein components, located within the sub‐endothelial layer of blood vessel walls, as well as to the tendency of platelets to aggregate or form conglomerates with leucocytes.^8^, ^9^, ^10^

Although studies have confirmed blood platelet hyper‐activation in RRMS, and have shown the role of these cells in the development of inflammation and autoimmune processes,^11^ there is relatively little information on blood platelet functioning in SPMS. However, for the last few years, the role of platelets in haemostatic mechanisms in the development of the SPMS has been the subject of intensive studies by our research team.^12^, ^13^, ^14^

The available data, including our previous studies, clearly indicate excessive intravascular activation of these cells and their hyper‐responsiveness to the number of physiological activators in SPMS. Intensified activation is manifested by, amongst other signs, an increase in adhesion properties, the formation of platelet aggregates and changes in the metabolism of blood platelets.

The latest epidemiological studies confirm the high risk of stroke or myocardial infarction in MS (especially in SPMS). That is, ischaemic events are directly associated with irregular platelet functions and their prothrombotic activity.^15^, ^16^ An analysis of over 6000 patient deaths in the Danish National Registry of MS patients revealed that death by vascular or cardiac disease was the most frequently listed cause of death outside of MS itself.^17^ Our existing findings state that platelet activation is an epiphenomenon consequent to the progression of MS, and probably secondary to endothelial injury, which causes the exposure of platelets to a variety of stimuli.^12^, ^13^, ^14^

A study scheduled by our research group will, for the first time, direct the search for the origins of the hyperactivity of platelets towards molecular changes within platelets in SPMS. Our studies now are aimed at understanding the molecular mechanisms of the clearly heightened prothrombotic activity of blood platelets in SPMS, by analysing their proteomes. For this current study, we conducted a comparative analysis of the platelet proteomes of SPMS patients and healthy controls, to demonstrate the quantitative and qualitative differences in the functional changes observed in SPMS.

## 2 MATERIALS AND METHODS

### 2.1 Chemicals

2‐[4‐(2‐hydroxyethyl)piperazin‐1‐yl]ethanesulfonic acid (HEPES), 3‐[(3‐cholamidopropyl)dimethylammonio]‐1‐propanesulfonate (CHAPS), Tris, KCl, glucose, iodoacetamide, Dithiothreitol (DTT) and trifluoroacetic acid (TFA) were all purchased from the Sigma‐Aldrich company (USA). Acetonitrile grade ULC/MS was obtained from Biosolve (Netherlands). H_4_H_2_PO_4_came from Acros Organic (Belgium). All other chemicals used were reagent‐grade products purchased from POCh (Gliwice, Poland).

### 2.2 Patient demographics and clinical characteristics

All blood samples were delivered from the Neurological Rehabilitation Division III General Hospital in Lodz, Poland. The study samples were obtained from 50 patients (male n = 22; female n = 28), suffering from the secondary progressive course of MS, diagnosed according to the revised McDonald criteria (MRI—criteria regarded as the gold standard for MS diagnosis, and a requirement for establishing the disease).^18^ The patients had previously been observed for 1 year, with SPMS ascertained as defined by Lublin et al.^19^, ^20^ SPMS can be recognized by the initial relapsing‐remitting phase, followed by the progression with or without incidental relapses, remissions and plateaux. The patients were under observation by the Neurorehabilitation Ward for 3 weeks, during which time they did not receive any immunostimulators, immunomodulators or hormones. Additionally, treatment with immunomodulating therapies had not been used in their progressive stage of MS for almost 1 year. This kind of inclusion criteria allowed us to avoid interference from the effects of these drugs on the examined parameters.

The clinical parameters of the selected SPMS patients were as follows: mean age 48.2 ± 15.2 years; mean disease duration 14.3 ± 8.3 years and body mass index 21.1 ± 9.7. The Kurtzke Expanded Disability Status Scale (EDSS scores) were 5.5 ± 1.8 (the EDSS scale ranges from 0 to 10 in 0.5 unit increments that represent higher levels of disability). EDSS is widely used and accepted as a valid tool for clinically measuring and evaluating MS patients’ levels of functioning. It is a means of quantifying the level of disability of eight functional systems under multiple sclerosis, and allows for standardization of neurostatus scoring. This helps neurologists to assess the functional condition of the patient. EDSS scores of 4.0 and above indicate some degree of gait impairment. Scores between 4.0 and 9.5 are determined by both walking ability and eight functional central venous system components (FS) scores.^21^

On the modified Rankin scale (mRS), patients ranged from 2 to 4 (between slight disability and moderately severe disability). The mRS is a clinician‐reported measure of global disability, widely applied in measuring the degree of dependence in the daily activities of patients receiving physical therapy.^22^

The Beck Depression Inventory (BDI scores) for the patients were 9.6 ± 4.6. BDI evaluation is one of the most widely used psychometric tests for measuring the severity of depression, and according to Beck et al^23^ is categorized into: normal (0‐13); mild (14‐19); moderate (20‐28) and severe depression (over 29).

The control human blood samples were collected from 50 healthy volunteers (man n = 19; female n = 31), not taking any medication, who had never been diagnosed with MS or other chronic diseases, and who at the time were without any neurological or hormonal illness, or any chronic inflammation. The control group and SPMS patients were matched by age and gender. These two populations were statistically compared, which confirmed the homology between them in terms of age and gender. The protocol and all procedures were conducted according to the Helsinki Declaration and were approved by the Bioethics Committee of the Medical University of Lodz, Poland, with Resolution No. RNN/260/08/KB.

### 2.3 Blood platelet isolation

The human blood samples were collected into S‐Monovette® CPDA_1_ (citrate phosphate, dextrose, adenine) tubes. All blood samples (control group and patients’) were drawn in the morning (8 am‐9 am) in fasting status, and stored using the same protocol. Blood was taken from the median cubital vein lying within the cubital fossa, anterior to the elbow. The blood platelets were isolated by differential centrifugation, as described by Wachowicz and Kustroń.^24^ The whole‐blood samples were centrifuged (200 *g*, 12 min, 37°C) to obtain platelet‐rich plasma. Next, three quarters of the top layer of platelet‐rich plasma was collected and return centrifuged (350 *g*, 15 min, 37°C). These demented platelets were washed and re‐suspended in modified Tyrode's (Ca^2+^/Mg^2+^) free buffer (127 mM NaCl, 2.7 mM KCl, 0.5 mM NaH_2_PO_4_, 12 mM NaHCO_3_, 5 mM HEPES, 5.6 mM glucose, and pH 7.4). The amount of platelets in the samples was estimated using the photometric method, according to Walkowiak et al.^25^ Isolated platelet samples were suspended in a lysis buffer (7 M urea, 2 M thiourea, 4% CHAPS, 30 mM Tris), and vortexed (five times for 30 s at 30 s intervals), for accurate destruction of the cellular structure. After collection of patients’ and controls’, all samples were coupled for each group.

### 2.4 2‐D Fluorescence difference gel electrophoresis (2D‐DIGE) electrophoresis

The first step included estimation of protein concentration in samples using a 2‐D Quant Kit (GE Healthcare). Next, the samples were cleansed of salt and dirt fragments from nucleic acids, phospholipids, lipids and polysaccharides using a 2‐D Clean‐Up Kit (GE Healthcare). After adjusting their pH to 8.5, purified proteins were labelled with fluorescent markers Cy3 and Cy5 (Dige Fluors herbicides, GE Healthcare). After labelling, the samples (15 µg of proteins) received Isoelectric focusing (IEF), followed by a 12‐h rehydration procedure at room temperature. The IEF process was performed on 7‐cm gel strips of a 3‐10 pH gradient (GE Healthcare), using an Ettan^TM^IPGphor3 IEF System (GE Healthcare) horizontal electrophoresis system, with a ceramic attachment Manifold. Separation parameters were set in accordance with the manufacturer's instructions: 0‐300 V for 30 min; 300‐1000 V for 30 min in gradient; 1000‐5000 V for 90 min in gradient and 5000 V for 30 min. The temperature of the cooling plate was 20°C. The total time of separation was 3 h (8 KVH). After this procedure, the focusing gel strips were balanced, first in a buffer containing 1% DTT (15 min), and then in a buffer with 2.5% iodoacetamide (15 min).

Separation according to molecular weight was carried out using SE 250 (GE Healthcare) horizontal electrophoresis apparatus. Focused, balanced gel strips were used with homogeneous 12.5% polyacrylamide gel (10 × 8 cm). Separation was carried out according to the manufacturer's protocol. The first stage separation parameters were 120 V, 20 mA, 30 W, and in the second stage 200 V, 50 mA, 30 W. A second separation stage was conducted until the front of the lower limit of the gel was reached. After this second separation, the gel was scanned with an Ettan^TM^ DIGE Imager (GE Healthcare) fluorescent scanner. Digital images of the obtained protein maps were analysed densitometrically using Image Master 2D Platinum software 6.0 (GE Healthcare), which allows for differences in the distribution of proteins in each sample to be seen. The first part of this analysis was detection of spots on the gels. After selecting the reference section for each group, the process of matching the spots within each group and determining the coefficients of the variation of peptides expression was conducted. Changes in the value of the expression coefficients from −1.4 to 1.4 were considered significant.

### 2.5 Mass spectrometry

Selected spots following excision from the 2D electrophoresis gel were reduced in 10 mM DTT, alkylated in the presence of 50 mM iodoacetamide and digested with trypsin (sequencing grade modified Trypsin 10 mg/ml, Promega), at 37°C for 14 h. The obtained peptides were extracted with 35 µl of 0.1% trifluoroacetic acid (TFA) and 2% acetonitrile, and applied to a C18 column (pre‐column nano Acquity UPLC, Waters). After elution with a gradient of acetonitrile in the presence of formic acid, peptides were seen (0.5‐1 µl, containing 50‐500 ng of protein) on an Anchor Chip plate (previously rinsed with 800 µl of isopropanol and water), then air‐dried at room temperature. Next, on a plate of samples, a 1 µl of α‐cyano‐4‐hydroxycinnamic acid (HCAA) matrix was added (1.4 mg of 4‐HCAA—Bruker Daltonics, Germany) was dissolved in a 2 ml diluent containing 85% acetonitrile, 0.1% TFA and 1 mM NH_4_H_2_PO_4_), and allowed to dry at room temperature. For the samples, a 0.5 µl calibrator (peptide calibration standard acc. to Bruker Daltonics, Germany), mixed with 1 µl of matrix was spotted and used for proper instrument calibration.

Matrix‐assisted laser desorption/ionization with time‐of‐flight mass spectrometry (MALDI‐TOF/TOF) imaging was performed with an Ultrafle Xtreme MALDI TOF/TOF (Bruker Daltonics, Germany), equipped with a smart beam laser at a 25%‐40% power setting. Mass spectra were processed using baseline correction and smoothing using Flex Analysis software (Bruker Daltonics, Germany). For each peptide, three peaks with the highest signal were selected for fragmentation. Identification of the peptides was made with a Brukers’ bioinformatics platform—the Protein Scape 3.0 (Bruker Daltonics, Germany), with the MASCOT search engine using the Swiss‐Prot Database.

## 3 RESULTS

**Figure 1:**
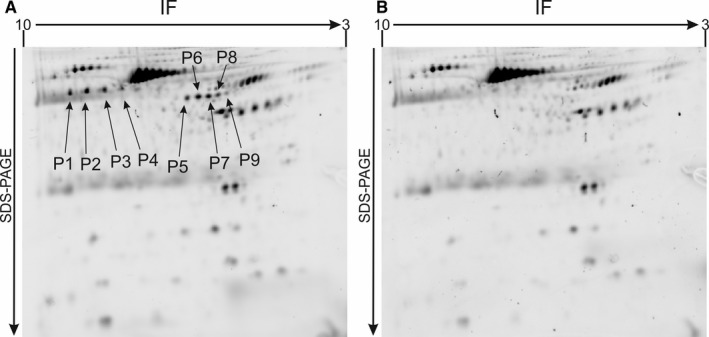
High‐resolution 2D‐DIGE electrophoretic patterns of blood platelet proteome obtained from SPMS patients (A), and the control group (B). Proteins were separated using isoelectric focusing (pH range 3‐10, 7 cm), and 12.5% SDS‐PAGE gels. This is a representative image of the 2D analysis. Spots highlighted as P1‐P9 have increased expression in SPMS platelets in comparison to the control group, and were selected for mass spectrometry analysis

To make a detailed assessment of the changes in the proteome of blood platelets in patients with SPMS, we performed two‐step analysis of the clinical material using 2‐D fluorescence difference gel electrophoresis (2D‐DIGE) and MALDI‐TOF/TOF mass spectrometry‐based proteomics. In the first step of our research, we performed a two‐dimensional, electrophoretic comparative separation of the blood platelet proteins obtained from the SPMS patients, and the healthy control group. During densitometric evaluation of the obtained digital images of protein maps, we observed differences between the electrophoretic patterns of SPMS platelets and the control samples (Figure 1). In gels with SPMS platelet proteins, additional groups of peptides were present. Next, we performed spot‐matching in gels (indicating the control platelets as a gel reference), to determine the peptide expression coefficient. The pixel volume of each spot was calculated, normalized and compared between the two groups using the Student's *t* test. For all the selected spots, the *P*‐values between the SPMS and control groups were <0.05. Of all the peptides identified in the platelet proteome, nine spots (P1‐P9 highlighted in Figure 1A) had significant up‐regulation in expression, relative to the control. The greatest increase of expression (coefficient = 6.2 ± 1.6) was obtained for spot P9, while spots P5‐P8 had expression coefficient values ranging from 4.2 to 3.4. Expression coefficients for peptides P1‐P4 were in the range of 1.8‐1.4. Detailed values for each spot are presented in Table 1.

**Table 1:** List of proteins identified by MALDI‐TOF/TOF in spots selected during 2D‐DIGE analysis

Spot number	Protein detected	Mean of spot expression coefficient ± SD	PI	Mass of protein (from database) [kDa]
P1	Fibrinogen beta chain	1.8 ± 0.5	9.3	55.9
P2	Fibrinogen beta chain	1.7 ± 0.4	9.3	55.9
P3	α‐2 macroglobulin	1.4 ± 0.2	6.0	163.2
P4	Septin‐14	1.7 ± 0.3	5.8	50.0
P5	Fibrinogen gamma chain	4.2 ± 1.6	5.3	51.5
P6	Fibrinogen gamma chain	3.7 ± 0.9	5.3	51.5
P7	Fibrinogen gamma chain	3.8 ± 1.3	5.3	51.5
P8	Fibrinogen gamma chain	3.4 ± 1.1	5.3	51.5
P9	tubulin β‐1 chain	6.2 ± 1.6	4.9	50.3

The table presents identified proteins with a mean of expression coefficient ± SD. All variants of the protein spots have a fold change cut‐off ≥1.4 and *P* < 0.05

For detailed characterization of the proteome changes in the blood platelets of the SPMS patients, in the next stage of our research we performed mass spectrometry analysis of the P1‐P9 spots. The differentially expressed protein spots were excised from the preparative gels, subjected to trypsin digestion, then sequenced using MALDI‐TOF/TOF spectrometry. All spots were successfully identified as known proteins in the SWISS‐PROT database. The results of the mass spectrometry analysis for the identified spots are listed in Table 1. Peptides P1 and P2 were characterized as a fibrinogen beta chain, P3 was α‐2 macroglobulin, P4 was septin‐14, spots P5‐P8 were derived from the fibrinogen gamma chain, and P9 from the tubulin β‐1 chain (Table 2).

**Table 2:** MS/MS data of identified spots P1‐P9

Spot number	Sequence coverage (%)	Sequences of unique peptides
P1	5.3	HQLYIDETVNSNIPTNLR and IRPFFPQQ
P2	5.3	HQLYIDETVNSNIPTNLR and IRPFFPQQ
P3	2.1	AIGYLNTGYQR; HYDGSYSTFGER and FQVDNNNR
P4	42.1	ENNIRCLTTIGHFGFECLPNQLVSR; CLTTIGHFGFECLPNQLVSRSIR; STLIDTLFNTNLK; LTVVETVGYGDQIDK; EASYQPIVDYIDAQFEAYLQEELK; SLFEYHDSR; SLFEYHDSRVHVCLYFISPTGHSLK; SLDLLTMKNLDSK; NLDSKVNIIPLIAK; NDLQTFKNK; QEFYDQCQREEEELK; FEHLKMIQQEEIR; MIQQEEIRK and MKAASEALQTQLSTDTK
P5	6.0	VELEDWNGR and CHAGHLNGVYYQGGTYSK
P6	6.0	YLQEIYNSNNQK and LTIGEGQQHHLGGAK
P7	3.3	VELEDWNGR and IIPFNR
P8	6.4	DNGIIWATWKTRW; GGAKQVR and HPAETEYDS
P9	14.6	EHGIDLAGSDR; AYGRKYVPR; LFQPDSFVHGNSGAGNNWAK; SLRFPGQLNADLRK and FAPLTAQGSQQYR

## 4 DISCUSSION

Blood platelets are a crucial element of the coagulation process and are involved in various pathologies, such as atherosclerosis and thrombosis. Due to the large number of specific membrane receptors, blood platelets are highly reactive cells, readily activated by many physiological and un‐physiological agonists.^26^, ^27^

In the human body, about 1 × 10^11^ platelets are created every day as a result of complex processes of differentiation, maturation and fragmentation of megakaryocytes.^28^, ^29^ It has long been known that platelet proteins can have different origins—some are synthesized in megakaryocytes, and some derive directly from blood plasma.^30^ However, studies completed in recent years have shown that un‐nucleated platelets are able to succeed protein synthesis. About 5000 mRNA transcripts have been detected in blood platelets, which represent half of all transcripts located in megakaryocytes.^31^ Additional blood platelets contain very stable mRNA transcripts with a long lifespan, correlated with platelet lifetime. This is very important, as the absence of a nucleus in these cells means they are unable to restore their mRNA pool.^32^

Proteomics can be a helpful tool in the search for biomarkers, in the diagnosis of rare platelet disorders or in the dissection of molecular mechanisms underlying a specific condition. Proteomic blood platelet analysis is probably the best tool, and an invaluable one, for characterizing the fundamental processes that affect platelet homeostasis, thus determining the roles of platelets in health and disease.^33^ Studies performed in the last few years have demonstrated that changes in blood platelet proteomes are associated with the risk of thrombotic events. The first studies that can be said to have initiated the field of platelet proteomics in clinical research were performed in 2008 by Arias‐Salgado et al.^34^ In that paper, the authors described blood platelets obtained from patients with arterial thrombosis having three decreased protein spots, and five increased protein spots, involved in cytoskeletal organization. Subsequent studies^35^, ^36^, ^37^ were performed on platelets obtained from patients with acute coronary syndrome/atherosclerosis, and demonstrated changes in the proteome of these cells. In blood platelets from patients with non‐ST‐segment elevation of acute coronary syndrome (NSTE‐ACS). Parguina et al^35^ observed a difference in the expression of 14 proteins involved in signalling or cytoskeletal functions, nine of which are known to participate in platelet activation by αIIbβ3 and/or GPVI receptors. In patients with ST‐segment elevation, myocardial infarction (STEMI) in platelet proteomes, a different expression of proteins was observed that were involved in actin cytoskeleton signalling, integrin signalling and integrin‐linked kinase signalling and activation by the collagen receptor GPVI.^36^ However, as a control group both studies’ authors used stable chronic ischaemic cardiopathy patients, who also had increased blood platelet activity and may have had changes in proteomes compared to healthy individuals. Banfi et al^37^ demonstrated changes in the proteome of platelets obtained from patients with stable or acute coronary atherosclerosis. This research group identified six differentially expressed proteins: two involved in energy metabolism, three associated with the cytoskeleton and one involved in protein degradation.

Our two‐dimensional differential gel electrophoresis and mass spectrometry‐based proteomic study indicated the increased presence of four proteins (fibrinogen, α‐2 macroglobulin, septin‐14 and tubulin β‐1 chain) in the proteome obtained from MS patients. According to the pro‐coagulant activity of platelets in MS, the most interesting of these seems to be the increased concentration of fibrinogen. The fibrinogen molecule is a large, soluble glycoprotein with a molecular weight of 340 kDa, containing in its structure three pairs of different polypeptide chains, identified as: Aα (610aa, 67 kDa); Bβ (461aa, 56 kDa) and γ (411aa, 48 kDa). These chains are connected by 29 disulfide bonds forming a dimeric molecule (Aα Bβγ)_2_.^38^ Fibrinogen is a key mediator in the formation of blood platelet aggregates, and the binding of fibrinogen to GPIIb‐IIIa on activated platelets results in platelet aggregation, presumably by crosslinking adjacent, activated platelets. Fibrinogen binds to GPIIb‐IIIa on agonist‐stimulated platelets with a dissociation constant (*Kd*) of approximately 100 nM, which is nearly 100 times less than the concentration of fibrinogen in plasma.^39^ Blood platelets contain three types of granules in their structure: dense granules, lambda granules and alpha granules. Activated platelets secrete the contents of these granules through their canalicular systems to the exterior environment. Fibrinogen is a part of the alpha granules’ content, and is realized during activation.^26^ These released molecules can play a major role in the formation of blood platelet aggregates. A study performed by Ang et al^40^ suggested that the plasma fibrinogen level increases blood platelet reactivity, and is a significant predictor of coronary ischaemic events.

There is relatively little information about blood platelets’ functioning in MS However, the available data, including from our own earlier studies,^12^, ^14^ clearly indicate that SPMS platelets are more sensitive to agonists and that their response is significantly stronger than that of platelets obtained from healthy patients. The mechanisms of increased platelet activation in MS also remain unknown. Our comparative analysis of the platelet proteome presented in this study demonstrated significant differences in the most important prothrombotic protein—fibrinogen, which seems to confirm the accuracy of, and potentially explain, our previous observations. Understanding the molecular mechanisms responsible for the increased platelet activation in MS could have important implications in the search for effective therapies and prevention of ischaemic events in this at‐risk group. This study provides new knowledge of the potential existence of the molecular mechanisms responsible for the acceleration of the platelet pro‐coagulant function in SPMS. This can help to identify new targets for therapy in the future, which could then be used not only in this stage of the disease. Due to the complexity of the pathological processes in MS, its medication must be multifaceted, and blood platelets are an important part of the pathogenesis of MS The next study should be focused on an explanation of the molecular pathway, leading to the increased concentration of fibrinogen in the blood platelets in SPMS. This analysis should include sequencing and expression of mRNA for platelet fibrinogen in patients with SPMS, as well as miRNA expression profiles, as these molecules are responsible for regulation of the mRNA translation process.

## CONFLICT OF INTEREST STATEMENT

The authors confirm no conflicts of interest.
